# Maternal age effects on fecundity and offspring egg‐to‐adult viability are not affected by mitochondrial haplotype

**DOI:** 10.1002/ece3.4516

**Published:** 2018-10-18

**Authors:** Rebecca E. Koch, James M. Phillips, M. Florencia Camus, Damian K. Dowling

**Affiliations:** ^1^ School of Biological Sciences Monash University Clayton Victoria Australia; ^2^ Department of Genetics, Evolution and Environment University College London UK

**Keywords:** aging, *Drosophila melanogaster*, evolution of aging, mitochondrial genome, mitonuclear, mother's curse, mtDNA, reproduction

## Abstract

While numerous studies have demonstrated that mitochondrial genetic variation can shape organismal phenotype, the level of contribution the mitochondrial genotype makes to life‐history phenotype across the life course remains unknown. Furthermore, a clear technical bias has emerged in studies of mitochondrial effects on reproduction, with many studies conducted on males, but few on females. Here, we apply a classic prediction of the evolutionary theory of aging to the mitochondrial genome, predicting the declining force of natural selection with age will have facilitated the accumulation of mtDNA mutations that confer late‐life effects on female reproductive performance. This should lead to increased levels of mitochondrial genetic variation on reproduction at later‐life stages. We tested this hypothesis using thirteen strains of *Drosophila melanogaster* that each possessed a different mitochondrial haplotype in an otherwise standard nuclear genetic background. We measured fecundity and egg‐to‐adult viability of females over five different age classes ranging from early to late life and quantified the survival of females throughout this time period. We found no significant variation across mitochondrial haplotypes for the reproductive traits, and no mitochondrial effect on the slope of decline in these traits with increasing age. However, we observed that flies that died earlier in the experiment experienced steeper declines in the reproductive traits prior to death, and we also identified maternal and grandparental age effects on the measured traits. These results suggest the mitochondrial variation does not make a key contribution to shaping the reproductive performance of females.

## 1 INTRODUCTION

Sequence variation in mitochondrial DNA (mtDNA) is a pervasive characteristic across eukaryotes, but the functional and evolutionary significance of much of this variation remains unclear (Dobler, Rogell, Budar, & Dowling, 2014; Dowling, Friberg, & Lindell, 2008; Galtier, Nabholz, Glémin, & Hurst, 2009). While evolutionary biologists traditionally assumed that mitochondrial genomes evolved under a neutral equilibrium model and that mtDNA variation would not affect phenotype, this assumption has been challenged by numerous studies that have shown that mitochondrial haplotypes commonly affect the expression of a broad range of phenotypic traits, including senescence, fertility, and longevity (Dobler et al., 2014; Dowling, 2014; Dowling et al., 2008; Galtier et al., 2009; Vaught & Dowling, 2018).

In particular, mitochondria are routinely thought to stand at the core of aging processes. The mitochondrial theory of aging predicts that somatic mutations will accumulate within mtDNA sequences with advancing age, causing cellular dysfunction that ultimately leads to symptoms of senescence (Balaban, Nemoto, & Finkel, 2005; Dowling & Simmons, 2009; Harman, 1972). At the same time, the mutation accumulation theory of aging predicts that the genome will be enriched for heritable mutations in the germline that confer later‐life effects, given that the force of natural selection decreases with increasing age following the peak of reproductive productivity (Charlesworth, 2001; Medawar, 1952; Reynolds et al., 2007). Integrated together, these two theories predict mutation accumulation within the mitochondria at two levels—somatic and germline—that together drive physiological senescence with advancing age; however, the latter level has received scant attention when applied to the mitochondrial genome. The key prediction of the mutation accumulation theory applied to the mitochondria is that selection should optimize the mitochondrial DNA sequence for optimal function in early life, but may fail to purge mtDNA mutations from the germline that are expressed only in later life. Compared to mutation accumulation in nuclear DNA, late‐onset germline mutations might be particularly likely to be enriched in the mitochondrial genome, given the high mutation rate of mtDNA and an often theorized diminished efficacy of selection in shaping the mtDNA sequence (Lynch & Blanchard, 1998; Lynch, Koskella, & Schaack, 2006; but see Cooper, Burrus, Ji, Hahn, & Montooth, 2015). Accordingly, different mtDNA lineages should accumulate different pools of late‐onset mutational variance, given that the number of mutations, the sites of mutation, the identities of nucleotides involved, and their associated magnitudes of effect will differ across lineages.

This concept of heritable mitochondrial mutation accumulation driving age‐related declines in performance remains largely untested. While there is evidence that somatic mitochondrial genomes harbor more mutations late in life, suggesting increased mitochondrial dysfunction and mutation rate with age (Bua et al., 2006; Hiona & Leeuwenburgh, 2008; Kujoth et al., 2005; Larsson, 2010; Wallace, 2010), tests for germline mutation accumulation have focused predominantly on the nuclear genome (Durham, Magwire, Stone, & Leips, 2014; Leips, Gilligan, & Mackay, 2006; Reynolds et al., 2007; Tatar, Promislow, Khazaeli, & Curtsinger, 1996). Research in model systems indicates that variation in mtDNA haplotype can influence longevity and other aging‐related parameters (Camus, Clancy, & Dowling, 2012; Camus, Wolf, Morrow, & Dowling, 2015; Dowling, Maklakov, Friberg, & Hailer, 2009; Dowling, Meerupati, & Arnqvist, 2010; Maklakov, Friberg, Dowling, Arnqvist, & Promislow, 2006; Rand, Fry, & Sheldahl, 2006; Zhu, Ingelmo, & Rand, 2014), but it is unclear whether these longevity‐modifying mtDNA variants are late‐onset in their expression (consistent with mutation accumulation theory) or expressed across the entire lifespan.

**Figure 1:**
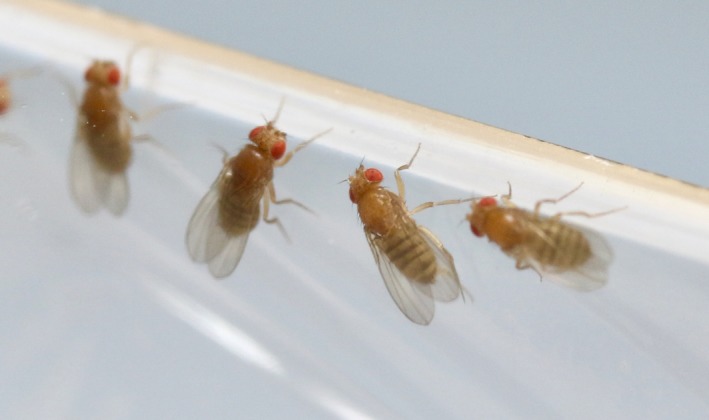
Wild‐type female fruit flies, *Drosophila melanogaster*

To redress these knowledge gaps, here we tested for variation in age‐related changes in female reproductive performance across naturally occurring mitochondrial haplotypes in the fruit fly *Drosophila melanogaster* (Figure 1). We focused our experiments on females for two reasons. Firstly, the mitochondria are maternally inherited, which means that natural selection on the mtDNA sequence can proceed through females only. Accordingly, if mutations appear in the mtDNA sequence that are benign or beneficial to females but harmful to males, selection may fail to eliminate them and they could contribute to the male‐specific mitochondrial genetic variance that acts across all life stages (Frank & Hurst, 1996; Gemmell, Metcalf, & Allendorf, 2004). This process has been termed Mother's Curse, and in support of this contention, several studies have documented male‐biased effects of mitochondrial mutations on life‐history phenotypes in fruit flies (Camus et al., 2012; Innocenti, Morrow, & Dowling, 2011; Patel et al., 2016; Xu, DeLuca, & O'Farrell, 2008), mice (Nakada et al., 2006), hares (Smith, Turbill, & Suchentrunk, 2009), and humans (Martikainen et al., 2017; Milot et al., 2017). By testing females, we assume that the mitochondrial genetic variation affecting our measured traits has been subjected to full force of natural selection at each given life stage. Secondly, we focus on females to help correct an emerging technical sex bias in the study of mitochondrial genetic effects on reproductive traits. A recent systematic review reported that female subjects are vastly underrepresented in studies of mitochondrial genetic effects on reproductive outcomes (Vaught & Dowling, 2018).

We therefore assessed fecundity, offspring egg‐to‐adult viability, and survival in female flies, each harboring one of 13 different mtDNA haplotypes in an isogenic nuclear background, at each of five distinct age classes, which allowed us to test for the role of mtDNA variation in the expression of these traits across the life course. This experimental design explicitly enabled us to test for signatures of mutation accumulation in the mitochondrial genome, consistent with the predictions of the evolutionary theory of aging. Specifically, we predicted that if mitochondrial haplotypes have accumulated mutations with negative late‐life effects, then levels of mitochondrial variation for fecundity and egg‐to‐adult viability should increase in later age classes.

## 2 MATERIALS AND METHODS

### 2.1 Study system

We used 13 strains of *D. melanogaster*, each characterized by a distinct mitochondrial haplotype in an otherwise isogenic nuclear background. The mtDNA haplotypes were originally sourced from distinct geographical regions from around the globe: Alstonville, Australia (Als), Barcelona, Spain (Bar), Brownsville, USA (Bro), Dahomey, Benin (Dah), Hawaii, USA (Haw), Israel (Isr), Japan (Jap), Madang, Papua New Guinea (Mad), Mysore, India (Mys), Oregon, USA (Ore), Puerto Montt, Chile (Pue), Sweden (Swe), or Zimbabwe (Zim). To create the strains, mitochondrial haplotypes from each of these regions were placed alongside an isogenic nuclear background (*w* ^*1118*^ strain, BloomingtonStock Number 5905) by Clancy (2008). We received these strains in 2007 and immediately created two duplicates of each mitochondrial strain, which we then maintained by backcrossing virgin females of each duplicate to males from the *w* ^*1118*^ strain each generation. Furthermore, to ensure continued isogenicity of the nuclear background, we propagated the *w* ^*1118*^ strain through just one full‐sibling pair each generation. This meant that any de novo mutations in the *w* ^*1118*^ nuclear background would either be swiftly eliminated or be fixed and immediately transferred into the nuclear backgrounds of each of the strain duplicates. At the time of the experiments described below, each mitochondrial strain had been subjected to at least 50 generations of backcrossing to the *w* ^*1118*^ strain. As a result, all flies within a mitochondrial strain duplicate possess a functionally identical mitochondrial sequence, and flies across all 13 mitochondrial haplotypes possess the same *w* ^*1118*^ nuclear DNA.

We note that this experimental design is highly controlled to allow us to home in on phenotypic effects that map unambiguously to the mitochondrial genome. One limitation of this design, however, is that by constraining our inferences to the one isogenic nuclear background (*w* ^*1118*^), we are unable to determine whether any detected mitochondrial genetic variation is sensitive to epistatic interactions with nuclear genetic variation. Our current study will therefore provide an important proof‐of‐principle of the applicability of the classic evolutionary theory of aging to the mitochondrial genome and serve as an important foundation for follow‐up studies that assess age‐related mitochondrial genetic effects across a broader array of nuclear genetic backgrounds.

## 3 EXPERIMENTAL PROCEDURE

All focal flies used within this experiment were reared within density‐controlled 10 dram vials (about 100 eggs per vial) on a potato‐dextrose agar medium and were maintained under a constant 25°C and 12‐hr light‐dark cycle. To control for parental age effects, the grandparents of the focal flies were 2 or 3 days old and the parents 1 or 2 days old at the time of reproduction. Because grandparental and parental ages were not evenly distributed across classes, we combined these into a composite categorical variable with three levels for statistical analyses (grandparental age of 2, parental age of 1; grandparental age of 2, parental age of 2; grandparental age of 3, parental age of 2).

Virgin focal female flies were collected within 6 hr of their eclosion into adulthood and maintained in individual vials. On day 2 of their adult lives, these females were provided with ad libitum access to yeast. On day 3, each focal female was exposed to 2–4‐day‐old “tester” males of the *w* ^*1118*^ isogenic strain for 24 hr, and then each female was moved to a separate “laying vial” for 20 hr. These laying vials each contained a plastic spoon filled with 1 ml of medium, stained black (Queen Black Food Color; 2 drops per 500 ml of medium) to increase visibility of white eggs. Following this 20‐hr period, each focal female was then transferred to a “holding” vial containing a small amount of fresh medium and subsequently transferred to a fresh holding vial every 48 hr until the next measurement period.

After the focal females were removed from the laying vials, we recorded the number of eggs oviposited on the black potato‐dextrose agar medium contained within the spoons. We used this value—number of eggs laid by each female in a 20‐hr period—as our estimate of “fecundity” for each female at each age. The medium on each of these spoons was then translocated to a fresh “rearing” vial, containing 6 ml of fresh medium, and stored at 25°C under a 12‐hr light‐dark cycle. After 13 days, we counted the number of adult flies that had emerged from each of these rearing vials. As the purpose of this count was to assess egg‐to‐adult viability (the proportion of adults that successfully eclosed) associated with a given set of eggs, dead adults were included in the count as long as no part of them remained within the pupal case. While we treat egg‐to‐adult viability of the maternal clutch as a trait corresponding to a reproducing female's performance, the likelihood of each egg to progress through the larval and pupal stages and successfully eclose is a product of both maternal effects and direct effects on the individual developing zygote. As maternal effects have been found to have a strong influence on egg‐to‐adult viability in *D. melanogaster* (Hercus & Hoffmann, 2000; Kern, Ackermann, Stearns, & Kawecki, 2001), we include it here as an indicator of focal female investment in each egg (the number of eggs laid by each female that converted into adult offspring). At the very least, this measurement offers an important counterpoint to our count of total number of eggs laid per ovipositioning period by providing information on how many of a female's eggs survive to adulthood (Berrigan, 1991), which is a more accurate measure of female fitness than fecundity data alone. As our measure of egg‐to‐adult viability relates to a discrete clutch of eggs laid over a 20‐hr period, we refer to it hereafter as “clutch viability.”

We repeated this process (female mating and oviposition followed by holding) an additional four times for each focal female, with each assay interspersed by approximately 8 days. We therefore assessed each focal female's clutch viability and fecundity at an adult age of 4, 12, 20, 28, and 35 days. We tested an average of 60 female flies per mitochondrial strain, divided among three experimental blocks, for a total of 850 focal flies at the start of the experiment. Each experimental block was separated in time by either one or two generations of *Drosophila* propagation (14–28 days). Given that mortality rate increases exponentially at around day 35 of adult life in these strains (Camus et al., 2012), many of our focal flies did not survive all five sampling points (only 450 of the initial focal flies lived to be sampled on day 35). Although measuring longevity was not a main goal of this experiment, we recorded the last measurement day at which a fly was observed to be alive. For simplicity, we refer to this value as “lifespan,” but it denotes an 8‐day window of time during which a fly died (e.g., a lifespan of “12” designates a fly that died between days 12 and 20).

### 3.1 Statistical analysis

We tested for relationships between clutch viability, mitochondrial haplotype, and focal female age with binomial mixed models, using the “glmer” function of the *lme4* package (logit link function; version 1.1‐14; Bates, Maechler, & Walker, 2015) in R (version 3.4.1; R Core Team, 2018). Our response variable for clutch viability was a binomial vector composed of the number of eclosed adults and the number of eggs that failed to result in eclosed adults, which together comprise egg‐to‐adult viability. For this analysis, we first excluded any data points in which the focal female laid fewer than 4 eggs, because egg‐to‐adult viability estimates become uninformative (inflated with values of 0 or 1) at very low fecundity (Supporting information Figure S1). We statistically centered focal female ages around 0 prior to analysis. We performed step‐wise model reduction, starting from a maximal model and sequentially dropping terms that explained the least variance. We used log‐likelihood ratio tests to examine whether dropping a particular term from the model resulted in a statistically significant change in model deviance. Our initial model included fixed effects of focal female age (4, 12, 20, 28, or 35 days), our composite measure of parental/grandparental age (1 and 2, 2 and 2, or 2 and 3 days), and fecundity as a covariate (the number of eggs laid during the 20‐hr ovipositioning period, or one “clutch”; to test for relationships between fecundity and clutch viability); random intercept effects of block number (3 blocks), female identity (850 females), and an observation‐level term that accounted for over‐dispersion; and, random slope effects of focal female age (at time of laying the clutch) within (a) mitochondrial haplotype, (b) mitochondrial strain duplicate, (c) lifespan, and (d) lifespan nested in mitochondrial haplotype; all four random slope effects had correlated random intercept effects. After model reduction, we retained several nonsignificant random intercept effects in our final model (mitochondrial haplotype, female identity, block, and mitochondrial strain duplicate) because these terms are essential to the structure of our data set. We assessed the significance of each term in the final model with log‐likelihood ratio tests and maximum likelihood estimation. Because our final binomial model resulted in a convergence warning from *lme4*, we also arcsine‐square‐root transformed the clutch viability data, modeled it using a linear mixed model (“lmer” function), and performed the same model reduction process, although without the observation‐level random effect. The final reduced model of the transformed data contained the same terms and was qualitatively similar to the final binomial model (Supporting information Table S1), substantiating the robustness of the results from the binomial models.

We then performed a similar process of model reduction to test for relationships between fecundity, mitochondrial haplotype, and female age using a generalized linear mixed model with a Poisson distribution in *lme4*. Our initial model contained the same terms as the initial model for the egg‐to‐adult viability analysis, except the number of eggs laid in a given time period (i.e., fecundity) was included as the response variable instead of as a fixed covariate. As before, we used log‐likelihood ratio tests and maximum likelihood estimation to evaluate the significance of effects included in the final model, and we retained several nonsignificant random effects (mitochondrial haplotype, female identity, block, and duplicate) to accurately reflect the hierarchical structure of the data set.

We next tested whether mitochondrial haplotype may have affected the lifespan of focal females using a survival analysis. We used the “coxme” function in the *coxme* package (version 2.2‐5; Therneau, 2015) in R (R Core Team, 2018) to build a mixed effects Cox proportional hazards model to test for effects of fecundity and parental/grand‐parental ages (as fixed effects) and mitochondrial haplotype, duplicate, block, and female identity (as random intercept effects) on likelihood to survive to the next measurement period. We censored the data to account for females still alive at the end of the experiment (day 35).

Lastly, we tested whether levels of mitochondrial genetic variation for clutch viability and fecundity varied over time (with increasing female age). We divided the data into five subsets based on focal female age, each isolating measurements from a particular age group (4, 12, 20, 28, or 35 days old). We then fitted each to a simplified binomial model that tested for effects of fecundity and parental/grand‐parental ages (as fixed effects) and mitochondrial haplotype, duplicate, block, and observation number (as random effects) on clutch viability. We repeated these models with fecundity instead of clutch viability, using simplified Poisson models of the same form (except removing number of eggs from the fixed effects). We then estimated the proportion of variance explained by mitochondrial haplotype at each of the five ages using formulae and R code described in Nakagawa and Schielzeth (2013) and Johnson (2014), and we used the “simulate” function within *lme4* to estimate ranges of possible values with bootstrapping.

## 4 RESULTS

**Figure 2:**
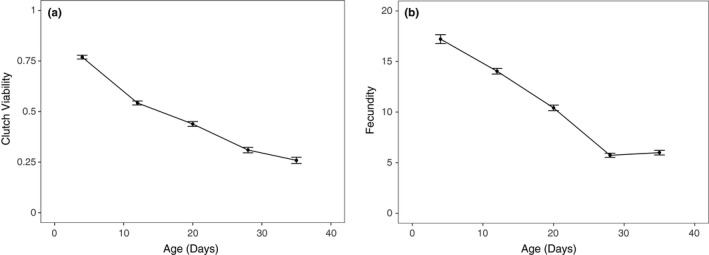
Declines (±*SE*) in average clutch viability (a) and fecundity (b; number of eggs laid in a 20‐hr period) with age

**Figure 3:**
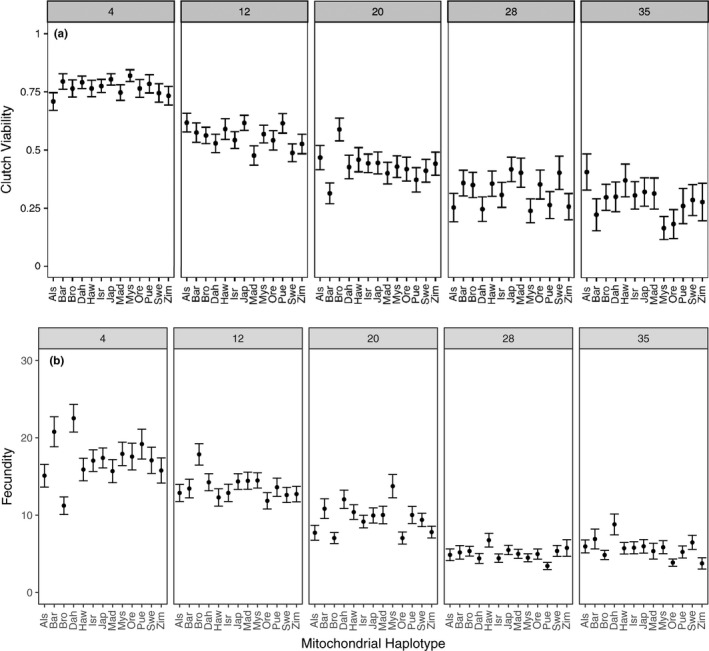
Average (±*SE*) clutch viability (a) and fecundity (b; number of eggs laid in a 20‐hr period) vary nonsignificantly among mitochondrial haplotypes, but decline with age. Panels depict the results from flies of different ages (4, 12, 20, 28, or 35 days, from left to right)

**Figure 4:**
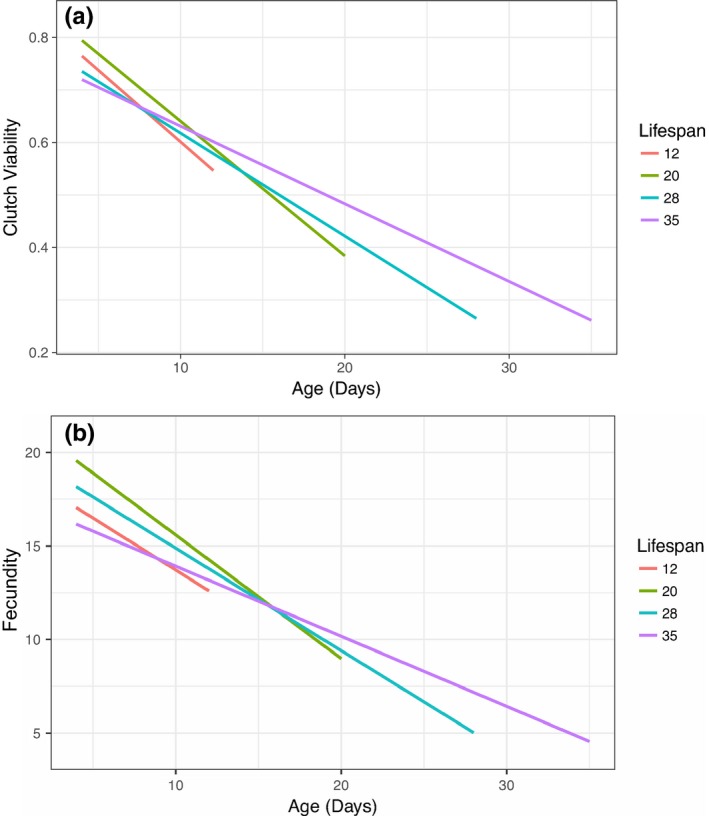
The slope of the decrease in clutch viability (a) or fecundity (b; number of eggs laid in a 20‐hr period) with age differs depending on the lifespan of the female. “Lifespan” refers to the time period of death of each female, such that the group “12” refers to females that died between the age of 12 and 20 days. Females in group “35” were alive at the end of the experiment. Females that died between age 4 and 12 days are not represented on this graph because they only lived through one measurement period

**Figure 5:**
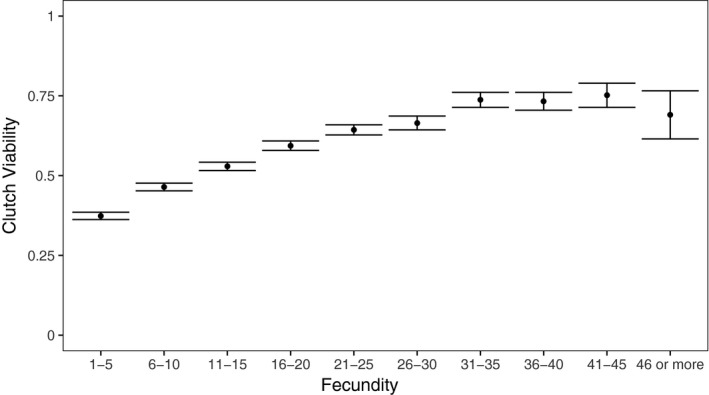
Average (±*SE*) clutch viability tends to increase with fecundity (i.e., larger “clutches” of eggs laid in a 20‐hr period tended to have higher rates of eclosion into adults)

We tested whether focal female age, mitochondrial haplotype, and their interaction influenced the clutch viability and fecundity in *Drosophila*. Our final model indicated that clutch viability decreased with age (Figure 2a), but mitochondrial haplotype had no significant effect on viability or the rate of decrease in viability with age (Figure 3a, Table 1). A significant random slope effect indicated that females that died earlier in the experiment also suffered a steeper decline in clutch viability before death (Figure 4a, Table 1). Our analysis of fecundity yielded similar results; older females had lower fecundity (Figure 2b), and females that died earlier in the experiment tended to have steeper declines in fecundity with age (Figure 4b, Table 2), but mitochondrial haplotype played little role in either relationship (Figure 3b, Table 1). These results suggest that if there is a mitochondrial genotypic effect on these traits, the effect size is small and remained undetectable at these sample sizes (an average of 60 females per haplotype). Lower clutch viability also was associated with lower fecundity (Figure 5, Table 1). The age at which each focal fly's parents and grandparents reproduced also influenced the results (Tables 1 and 2, Supporting information Figure S2).

**Table 1:** Statistical results of the generalized linear mixed model (binomial error distribution) after model reduction for predictors of clutch viability. This model fits a dataset that excludes exceptionally small clutches (number of eggs < 4). “L‐R” refers to results of log‐likelihood ratio tests (see Methods)

	Effect	Estimate	*SE*	L‐R *χ* ^2^	*p*
Fixed effects	Intercept	0.036	0.156		
	Age	−0.126	0.014	15.226	<0.001
	Fecundity	0.010	0.004	6.713	0.010
	Parent age of 2, grandparent age of 2	−0.330	0.090	8.325^a^	0.016
	Parent age of 2, grandparent age of 3	−0.024	0.121		

		Variance	L‐R *χ* ^2^	*p*
Random effects	Mitochondrial haplotype	0.003	0.177	0.674
	Lifespan	0.042	25.79^a^	<0.001
	Age by lifespan random slope	0.001	29.48^a^	<0.001
	Female ID	0.046	0.865	0.353
	Duplicate	1.38E‐8	0.002	0.965
	Block	1.48E‐8	0.0002	0.988
	Observation number	1.760	2460	<0.001

a df = 2; all other df = 1.

**Table 2:** Statistical results of the generalized linear mixed model (Poisson error distribution) after model reduction for predictors of fecundity (number of eggs laid in a 20‐hr period). The final model is the same as that for egg‐to‐adult viability except only the random intercept effect of lifespan was dropped as nonsignificant (*p *=* *0.162). “L‐R” refers to results of log‐likelihood ratio tests (see Methods)

	Effect	Estimate	*SE*	L‐R *χ* ^2^	*p*
Fixed effects	Intercept	2.386	0.109		
	Age	−0.039	0.006	13.325	<0.001
	Parent age of 2, grandparent age of 2	−0.281	0.124	67.347^a^	<0.001
	Parent age of 2, grandparent age of 3	−0.485	0.057		

		Variance	L‐R *χ* ^2^	*p*
Random effects	Mitochondrial haplotype	3.852E‐8	0	1
	Age by lifespan random slope	8.248E‐5	69.104	<0.001
	Female ID	0.0507	22.336	<0.001
	Duplicate	0.00461	2.419	0.12
	Block	0.00799	23.75	<0.001
	Observation number	0.433	4676.9	<0.001

a df = 2; all other df = 1.

Our survival analyses found that the only factor that was associated with lifespan was fecundity (Table 3). This relationship was positive, indicating that females who laid more eggs in a given time period were more likely to survive to the next measurement period.

**Table 3:** Statistical results of the random effects Cox proportional hazards model

	Effect	Estimate	*SE*	*Z*	*p*
Fixed effects	Fecundity	0.0169	0.00823	2.06	0.04
	Parent age of 2, grandparent age of 2	−0.406	0.717	−0.57	0.57
	Parent age of 2, grandparent age of 3	−0.0743	0.371	−0.20	0.84

		Variance
Random effects	Mitochondrial haplotype	0.000538
	Duplicate	0.195
	Block	0.284
	Fly identity	7.757

We found no evidence that mitochondrial haplotype explained a significant proportion of variance in clutch viability or fecundity within any single age class (less than 0.3% of total variance in any age class, within range of bootstrapped values; Supporting information Tables S2 and S3).

## 5 DISCUSSION

Here, we tested for effects of mitochondrial haplotype variation on female fecundity, clutch viability, and age‐related declines in these traits, as well as on a proxy of female lifespan. We observed a decrease in both fecundity and viability with increasing female age, which is consistent with previous findings in *D. melanogaster* (reviewed in Miller et al., 2014). However, we found no statistically significant effects of mitochondrial genetic variation on any of the parameters examined, indicating that mtDNA variation does not play a sizable role in contributing to the expression of these traits or in modifying the rate at which the traits exhibit senescence with advancing age in female *D. melanogaster*. Our results therefore suggest that mutation accumulation in the mitochondrial genome is unlikely to play a major role in mediating age‐related declines in reproductive performance in this species, at least in females. Instead, we found an interaction between our indicative measure of lifespan and age‐related declines in the reproductive traits; females that died earlier in the experiment experienced steeper declines in both clutch viability and fecundity leading up to their deaths.

Differential mortality in captive experimental studies may be an important, but often overlooked, source of variation in the analysis and interpretation of experimental studies. Even though we did not design our experiment to focus specifically on longevity, our results demonstrate that variation in lifespan could be predicted by the rate of physiological decline in female reproductive performance. It is particularly interesting that none of our main experimental sources of variation—mitochondrial haplotype, parental/grand‐parental ages, duplicate number, or block number—appeared to contribute to variation in lifespan. However, flies that produced more eggs in a given time period were more likely to survive to the next, which suggests that high fecundity was an indicator of individual physiological quality rather than a costly behavior tied to early mortality.

That we found no evidence of mtDNA contributing to variation in the rate of female reproductive trait senescence is consistent with recent findings that suggest that purifying selection is highly efficient at shaping the mtDNA sequence through females (Cooper et al., 2015; Popadin, Nikolaev, Junier, Baranova, & Antonarakis, 2013). Indeed, a systematic review of effects of mtDNA variation on components of male and female reproductive success across metazoans revealed male biases, suggesting that purifying selection on mtDNA tends to remove functional variation that negatively affects females (Vaught & Dowling, 2018). Accordingly, previous studies that leveraged these same 13 strains of flies have demonstrated that males, but not females, vary among mitochondrial haplotypes in longevity and rate of senescence (Camus et al., 2012, 2015), and expression of genes tied to reproductive function (Innocenti et al., 2011). Our results are generally consistent with these findings, given we did not detect a significant mitochondrial genetic effect on survival nor physiological aging in female flies.

However, a recent study by Camus and Dowling (2018) included similar measures of clutch viability and fecundity as those used in this study, but did find a significant effect of mitochondrial genetic variation on early life clutch viability (although not fecundity). Furthermore, they reported that intersexual genetic correlations across mtDNA haplotypes for these reproductive traits were generally negative, such that haplotypes that conferred greatest performance in females also conferred the lowest performance in males. This suggests that functional genetic variation in mitochondrial DNA has accrued under positive selection in females, despite costs to males.

It is unclear why Camus and Dowling's (2018) study of these fly strains found a significant effect of mitochondrial variation on clutch viability in 4‐day‐old female flies, but our current study found no such effect in females of any age group. Firstly, it is possible that the differences in significance between the two studies are due to sample size; Camus and Dowling (2018) tested about 400 more females at day 4 than we did in the current study. However, the patterns of variation we observed among mitochondrial haplotypes in this study (Figure 2) exhibit no trend toward the same patterns as presented in Camus and Dowling (2018), so it is more likely that methodological rather than statistical variation is responsible for the differences in our results.

Indeed, several differences in the methods of these two studies may have altered the physiological state of the experimental females and led to discrepancies. One of the key differences was the timing at which live yeast (the primary source of dietary protein) was provided to females. Females in the Camus and Dowling (2018) study were provided with yeast 24 hr earlier than in our study and therefore had more time to convert the dietary protein to resourcing of the oocytes. A second difference between studies is in the mating environment to which the experimental females were subjected. Each female was placed with two males for 24 hr in the current study, but one male for 12 hr in Camus and Dowling (2018). This is likely to have affected the remating rate and levels of sexual harassment experienced by females across the two studies, factors known to confer both direct and transgenerational consequences on female life‐history trait expression (Dowling, Williams, & Garcia‐Gonzalez, 2014; Garcia‐Gonzalez & Dowling, 2015; Garcia‐Gonzalez & Simmons, 2010; Priest, Galloway, & Roach, 2008). Finally, other minor differences in the experimental procedures between studies would alter the microenvironments within the vials in which females resides, such as the color of dye used to tint the food. Perhaps due to these methodological differences, average clutch viability of 4‐day‐old females in our study was higher (about 75% viability, on average) and average fecundity was slightly lower (about 17 eggs laid over 20 hr, or 0.85 eggs/hr) than those reported for 4‐day‐old females in Camus and Dowling (2018; about 50% viability and 23 eggs over 24 hr, or 0.96 eggs/hr). Young females in our study therefore laid fewer eggs over a slightly shorter ovipositional period than those in Camus and Dowling (2018), but offspring from those eggs were ultimately more likely to eclose into adults.

Regardless, the patterns of sexual antagonism across haplotypes revealed in Camus and Dowling (2018) and the discrepancy between their results and those of the current study suggest that further research is needed to assess the modes and mechanisms by which functional variation accumulates within the mitochondrial genome, and to understand the conditions under which this mitochondrial variation is expressed. Several studies have now revealed that gene‐by‐environment interactions extend to variation within the mitochondrial genome. Reaction norms associated with different haplotypes change across extrinsic factors, such as temperature, diet, and mating environment (Arnqvist et al., 2010; Dean, Lemos, & Dowling, 2015; Dowling, Abiega, & Arnqvist, 2007; Dowling et al., 2010; Hoekstra, Siddiq, & Montooth, 2013; Zhu et al., 2014), and intrinsic factors, such as age (Wolff et al., 2016), as well as across nuclear genetic backgrounds (Clancy, 2008; Mossman, Biancani, Zhu, & Rand, 2016). There is a need for further studies exploring mitochondrial genetic plasticity for reproductive success across environmental, physiological, and nuclear genetic contexts. This need is further underscored by the sex bias that currently exists in the study of mitochondrial genetic variation on reproductive traits, with relatively few data available on female trait expression (Vaught & Dowling, 2018).

In sum, our results suggest that mutation accumulation in the mitochondrial genome is not a key driver of reproductive trait senescence in females. Indeed, we did not uncover mitochondrial genetic variation affecting any of the traits measured in this study, a finding that reinforces the emerging evidence that functional effects of mitochondrial genetic variation are often male‐biased (Vaught & Dowling, 2018). Notwithstanding, we currently have a poor understanding for how mitochondrial genetic effects on organismal life histories will manifest across different ecological and nuclear genetic contexts, and when it comes to studies of mitochondrial effects on reproductive trait expression, there is a lack of studies investigating effects in females. Future research should address these knowledge gaps and extend the taxonomic breadth of studies by addressing these questions in new study systems to substantiate the broad applicability of these findings beyond the *Drosophila* model system.

## DATA ACCESSIBILITY

The dataset used in this study is available through Figshare (https://doi.org/10.26180/5b725e60621c5).

## AUTHOR CONTRIBUTIONS

R.K. assembled and analyzed the data, wrote the initial manuscript draft, and contributed to revisions. J.P. and M.F.C. contributed to initial project design, collected the data, and performed preliminary analyses. D.D. contributed to project design, supervised data collection, and contributed to data analysis and manuscript revisions.
