# Supplementary material for: Maternal age effects on fecundity and offspring egg‐to‐adult viability are not affected by mitochondrial haplotype
Source: Ecol Evol. 2018 Oct 18;8(22):10722–32. doi: 10.1002/ece3.4516 (PMC6262919; doi:10.1002/ece3.4516)
Supplement: Supplementary file 1 [file ECE3-8-10722-s001.docx]

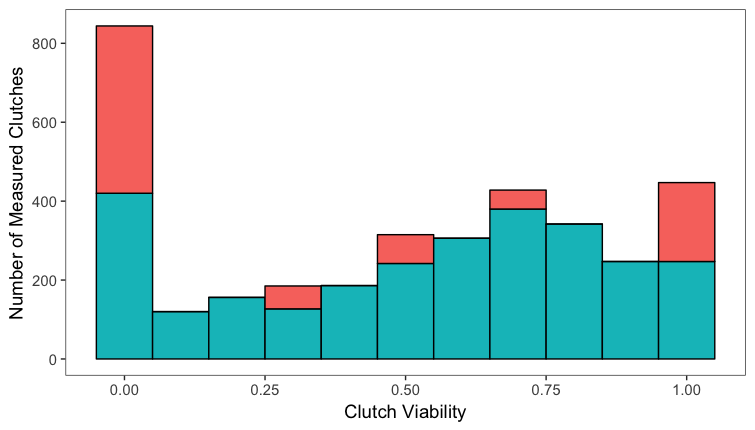


**Fig. S1** Histogram depicting the spread of clutch viability measurements in our dataset. Colors represent the size of each measured clutch: the orange portion of each bar indicates clutches that contained fewer than 4 eggs (laid within one 20-hour period), which were excluded from analyses of clutch viability. These small clutch sizes resulted in a disproportionate number of viability measures of 0 or 1.

**Fig. S2** Variations in the average clutch viability (a) and fecundity (b; number of eggs laid in each 20-hour period) of females with parents and grandparents that reproduced at different ages (parents aged 1 or 2 days, grandparents aged 2 or 3 days).

|  | **Effect** | Estimate | SE | L-R **𝜒^2^** | *P* |
| --- | --- | --- | --- | --- | --- |
| Fixed effects | Intercept | 0.765 | 0.038 |  |  |
|  | Age | -0.028 | 0.003 | 15.39 | <0.001 |
|  | Fecundity | 0.003 | 0.001 | 7.78 | 0.005 |
|  | Parent age of 2, grandparent age of 2 | -0.059 | 0.024 | 5.92* | 0.052 |
|  | Parent age of 2, grandparent age of 3 | 0.009 | 0.029 |  |  |
|  |  |  |  |  |  |
|  |  | Variance |  | L-R **𝜒^2^** | *P* |
| Random effects | Mitochondrial haplotype | 4.38E-04 |  | 0.447 | 0.504 |
|  | Lifespan | 0.003 |  | 19.34* | <0.001 |
|  | Age by lifespan random slope | 3.68E-05 |  | 23.91* | <0.001 |
|  | Female ID | 0.004 |  | 2.24 | 0.135 |
|  | Duplicate | 0 |  | 0 | 1 |
|  | Residual | 0.137 |  |  |  |

*df=2; all other df=1

**Table S1** Statistical results of linear mixed model after model reduction for predictors of egg-to-adult viability, using transformed data: clutch viability was transformed via the arcsine square root transformation for linearity. “L-R” refers to results of log-likelihood ratio tests (see Methods).

|  |  | Day 4 | | Day 12 | | Day 20 | | Day 28 | | Day 35 | |
| --- | --- | --- | --- | --- | --- | --- | --- | --- | --- | --- | --- |
|  | Effect | Estimate | SE | Estimate | SE | Estimate | SE | Estimate | SE | Estimate | SE |
| Fixed effects | Fecundity | 0.00618 | 0.00422 | 0.00715 | 0.00713 | 0.0243 | 0.0129 | 0.0277 | 0.0253 | 0.0565 | 0.0340 |
|  | Parent age of 2, grandparent age of 2 | -0.808 | 0.124 | -0.170 | 0.313 | 0.0144 | 0.225 | -0.0166 | 0.310 | 0.849 | 0.437 |
|  | Parent age of 2, grandparent age of 3 | -0.144 | 0.166 | -0.0860 | 0.196 | 0.696 | 0.344 | 0.0271 | 0.418 | -0.904 | 0.644 |
|  |  |  |  |  |  |  |  |  |  |  |  |
|  |  | Variance |  | Variance |  | Variance |  | Variance |  | Variance |  |
| Random effects | Mitochondrial haplotype | 4.827E-9 |  | 0.0168 |  | 0.0204 |  | 0 |  | 2.560E-6 |  |
|  | Duplicate | 3.092E-10 |  | 1.044E-10 |  | 0.0444 |  | 7.833E-10 |  | 3.593E-7 |  |
|  | Block | 3.534E-8 |  | 0.0489 |  | 6.480E-9 |  | 1.060E-9 |  | 1.343E-8 |  |
|  | Observation | 0.876 |  | 1.634 |  | 2.471 |  | 1.997 |  | 3.330 |  |
|  |  |  | |  | |  | |  | |  | |
| Proportion of total variance explained by mitochondrial haplotype (range of boostrapped values) |  | 1.12E-9 (0-0.002) | | 2.54E-3 (0-0.011) | | 2.44E-3 (0-0.020) | | 0 (0-0.018) | | 2.48E-7 (0-0.021) | |
| Significance of mitochondrial haplotype (L-R **𝜒^2^** (*P*)) |  | 0  (*P* = 1.00) | | 0.649  (*P* = 0.42) | | 0.099 (*P* = 0.75) | | 0 (*P* = 1) | | 0 (*P* = 1) | |

**Table S2** Statistical results of generalized linear mixed models (binomial error distribution) of clutch viability, broken down by age. We also report the proportion of total model variance explained by mitochondrial haplotype within each age group, the range of possible values for the proportion of total variance explained by mitochondrial haplotype (from bootstrapping), and the L-R significance of the mitochondrial haplotype term (evaluated by model comparison).

|  |  | Day 4 | | Day 12 | | Day 20 | | Day 28 | | Day 35 | |
| --- | --- | --- | --- | --- | --- | --- | --- | --- | --- | --- | --- |
|  | Effect | Estimate | SE | Estimate | SE | Estimate | SE | Estimate | SE | Estimate | SE |
| Fixed effects | Parent age of 2, grandparent age of 2 | -0.459 | 0.544 | -0.081 | 0.107 | -0.499 | 0.085 | 0.057 | 0.106 | -0.350 | 0.299 |
|  | Parent age of 2, grandparent age of 3 | -0.582 | 0.091 | -0.078 | 0.088 | -1.091 | 0.108 | -0.134 | 0.137 | -0.509 | 0.152 |
|  |  |  |  |  |  |  |  |  |  |  |  |
|  |  | Variance |  | Variance |  | Variance |  | Variance |  | Variance |  |
|  | Mitochondrial haplotype | 7.783E-10 |  | 2.44E-9 |  | 0.003 |  | 1.567E-10 |  | 0.003 |  |
| Random effects | Duplicate | 0.016 |  | 0.016 |  | 0.004 |  | 1.898E-10 |  | 0.014 |  |
|  | Block | 0.194 |  | 0.004 |  | 7.675E-4 |  | 0 |  | 0.050 |  |
|  | Observation | 0.456 |  | 0.371 |  | 0.383 |  | 0.396 |  | 0.050 |  |
|  |  |  |  |  |  |  |  |  |  |  |  |
|  |  | 6.33E-10 (0-0.012) | | 2.92E-9 (0-0.011) | | 3.72E-3 (0-0.009) | | 1.806E-10 (0-0.006) | | 3.24E-3 (0-0.021) | |
| Proportion of total variance explained by mitochondrial haplotype (range of boostrapped values) |  | 0  (*P* = 1.00) | | 0  (*P* = 1.00) | | 0.160 (*P* = 0.69) | | 0 (*P* = 1) | | 0 (*P* = 1) | |
| Significance of mitochondrial haplotype (L-R **𝜒^2^** (*P*)) |  |  | |  | |  | |  | |  | |

**Table S3** Statistical results of generalized linear mixed models (poisson error distribution) of fecundity (number of eggs per 20-hour laying period), broken down by age. We also report the proportion of total model variance explained by mitochondrial haplotype within each age group, the range of possible values for the proportion of total variance explained by mitochondrial haplotype (from bootstrapping), and the L-R significance of the mitochondrial haplotype term (evaluated by model comparison).
